# Constructing the magnetic bifunctional graphene/titania nanosheet-based composite photocatalysts for enhanced visible-light photodegradation of MB and electrochemical ORR from polluted water

**DOI:** 10.1038/s41598-017-12504-2

**Published:** 2017-09-25

**Authors:** Qian Zhang, Yihe Zhang, Zilin Meng, Wangshu Tong, Xuelian Yu, Qi An

**Affiliations:** 1 Beijing Key Laboratory of Materials Utilization of Nonmetallic Minerals and Solid Wastes, National Laboratory of Mineral Materials, School of Materials Science and Technology, China University of Geosciences, Beijing, 100083 China; 2 School of Resources and Environmental Engineering, Shandong University of Technology, Zibo, 255049 China

## Abstract

Photocatalysis is a promising strategy to address the global environmental and energy challenges. However, the studies on the application of the photocatalytically degraded dye-polluted water and the multi-purpose use of one type of catalyst have remained sparse. In this report, we try to demonstrate a concept of multiple and cyclic application of materials and resources in environmentally relevant catalyst reactions. A magnetic composite catalyst prepared from exfoliated titania nanosheets, graphene, the magnetic iron oxide nanoparticles, and a polyelectrolyte enabled such a cyclic application. The composite catalyst decomposed a methylene blue-polluted water under visible light, and then the catalyst was collected and removed from the treated water using a magnet. The photocatalytically treated water was then used to prepare the electrolyte in electrochemical reductive reactions and presented superior electrochemical performance compared with the dye-polluted water. The composite catalyst was once again used as the cathode catalyst in the electrochemical reaction. Each component in the composite catalyst was indispensable in its catalytic activity, but each component played different roles in the photochemical, magnetic recycling, and electrochemical processes. We expect the report inspire the study on the multi-functional catalyst and cyclic use of the catalytically cleaned water, which should contribute for the environmental and energy remedy from a novel perspective.

## Introduction

Environmental remedy and energy crisis call for scientific contributions with ever increasing urgency^1–5^. Organically polluted water, closely related with printing and dyeing industry, is a frequent side product of improved human life and civilization. Not only effective pollution decomposition but also appropriate applications of the treated water should contribute to the remedy of waste water. In addition, cyclic and multiple uses of one material for different purposes will also decrease the manufacturing demands and contribute to environmental protection and energy savings^6–10^. Although the catalytic decomposition of organic pollutions in water has been extensively studied^11^, the cyclic use of the treated water and the multi-purpose applications of the catalyst have remained under study.

To explain in detail, special attentions have been paid to developing effective strategies in degrading organic pollutants in water^12–16^. Visible light photocatalysts have been anticipated to be the promising solution to degrade organic dyes in water, and methods that enhance catalysts’ visible light catalytic power and operability have been heavily pursued after^17–20^. Semiconductor photocatalysts have drawn substantial attentions because of their appropriate electronic band structures and their abundance on earth. Previously we specifically focused on 2-dimensional exfoliated titania nanosheets (ETNs) for the following reasons: i) ETNs present an energy gap 0.6 eV larger than bulk anatase TiO_2_, promising their superior catalytic power^21^; ii) ETNs offer larger specific surface area and thus more catalytic active sites^22^; and iii) the high surface charge and small volume made ETNs easily processed using solution assembly techniques^23,24^. One big challenge in bringing ETNs a step closer to applications is to induce visible light activity to ETN-based catalysts. Several strategies have been proposed to address the visible light activity by combining with visible-light active component, including forming composite particles with narrow-bandgap semiconductor (CdS)^25,26^, noble metal (Pt, Au)^27–29^, or visible light responsive materials (Zn-Cr LDHsor CrOx)^24,30^, as well as elemental doping (with nitrogen or manganese)^31,32^. Another challenge in applying ETN-based catalyst would be enhancing its recovery ability. Traditionally, recycling of powder photocatalysts in waste solution can avoid the secondary contamination and reduce actual implementation cost, but it is a seriously difficult task due to its small particle size and high environmental temperature^33,34^. To prepare magnetic catalyst has been widely recognized as effective in removing the catalysts after the reaction and recycling catalysts for multiple time applications^35–38^.

Reports on the applications of the photocatalytically treated water remain sparse. It’s still doubtable how the photocatalytically cleaned water can be used. Due to the existence of residue organic species such as benzothiazole, azure A, maleic acid and other inorganic species such as NO_3_^−^, Cl^−^ and SO_4_^2−^, the degraded water is still harmful to drink by human and animals. However, the application of the degraded water in the industrial application might be a viable solution. In developing countries, both the resource of clean water and electric power are sparse. The conversion of polluted water into electricity will impose high potential in addressing both the environmental and energy crisis in these areas. A fuel cell is a promising strategy in exploring the redox active species in environments. The wide varieties of applicable redox species range from the mineral chemicals in oceans^39,40^, oxygen^41^, and bacterial metabolic systems^42^. Because of the diversity of the potentially applicable redox active species in fuel cells, we want to investigate whether the dye-polluted water, before and after photocatalytic treatment, can be used efficiently as the solvent in reductive reactions of the water-dissolved species and oxygen reduction reactions (ORR), which is a widely studied fuel cell anode reaction.

**Figure 1:**
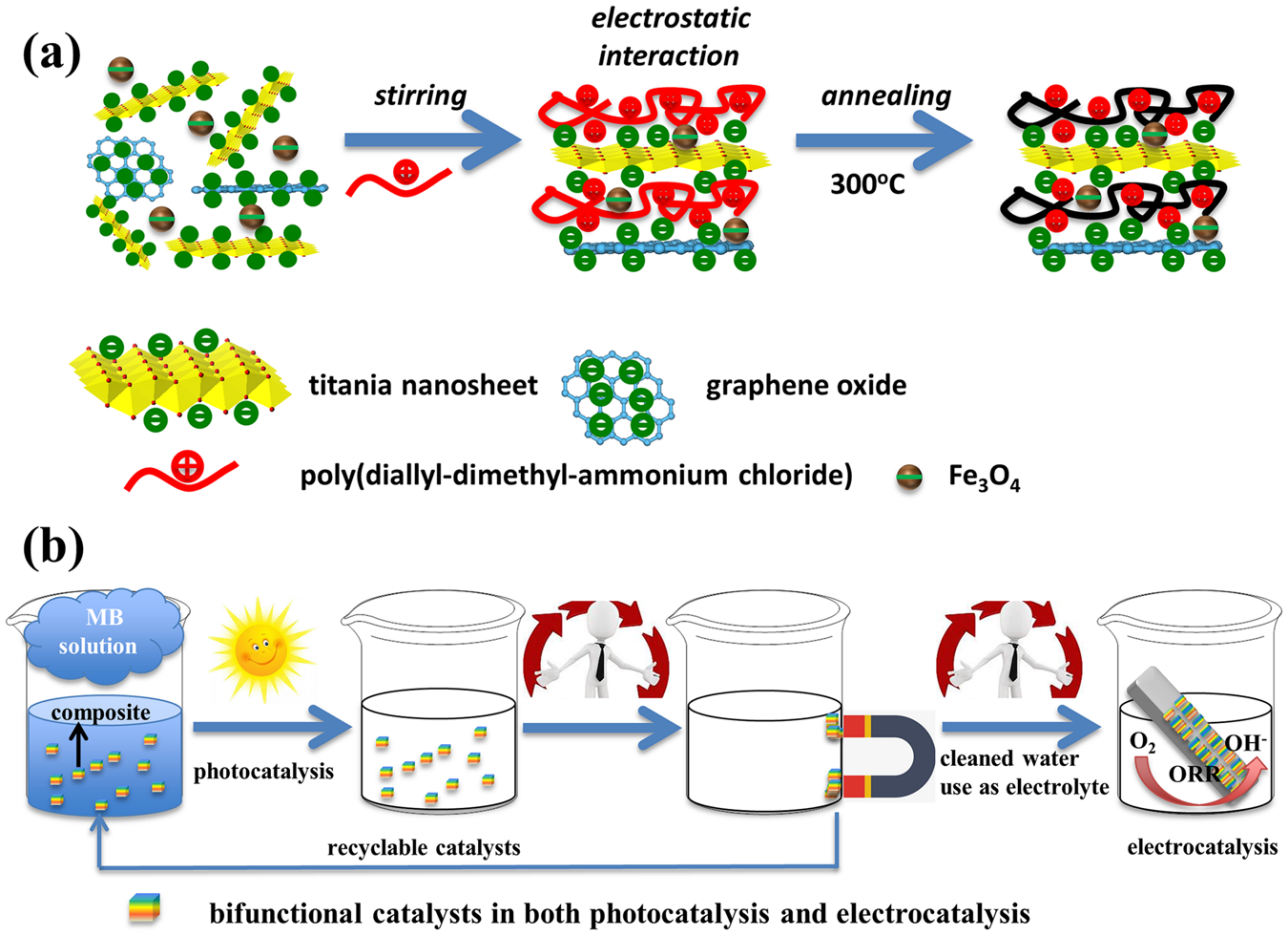
(**a**) The fabrication procedures of the magnetic composite photocatalyst and (**b**) schematic illustration of recyclable application of the photocatalytically cleaned water and the catalyst: MB-polluted water was first treated using the visible-light photocatalyst to decompose the organic dyes; subsequently the magnetic catalyst was collected using a magnet and the cleaned water was further used as an ORR solvent; the catalyst was also used as the electrocatalyst in the ORR.

In this report, we propose a concept of environmentally economic use of a bifunctional catalyst and also the cyclic use of the photocatalytically cleaned dye-polluted water in electrochemical electrolyte. The multiple and cyclic application of the catalyst and the treated water depends on the achievement of a magnetic catalytic material which functions as simultaneously an effective visible light photocatalyst and an ORR active electrocatalyst, prepared using ETNs, reduced graphene oxide, iron oxide nanoparticles, and a polyelectrolyte PDDA (Fig. 1). Because of the effective photodecomposition and magnetic capabilities of the catalyst, the photochemically cleaned water can be used as an appropriate solvent for ORR reaction. In addition, the catalyst prepared also possessed electrocatalytic activities, and can be used in both photocatalytic water treatment and ORRs. The catalyst was facilely prepared using a flocculation followed by a calcination process. Each component was indispensable in achieving the effective catalytic power, but the component each played different roles in the photocatalysis, magnetic recycling, and electrocatalysis processes. We hope that our report demonstrates a concept of designing multiple functional materials, which should hold high promise in recyclable applications of natural resources, including chemical products and polluted water.

## Experimental

### Materials

Poly (diallyl-dimethyl-ammonium chloride) (PDDA) was purchased from Sigma-Aldrich Tech Co. Ltd. TiO_2_ was provided by Aladdin Reagent Company. K_2_CO_3_, LiCO_3_, HCl, MoO_3_, NH_3_·H_2_O, FeCl_3_·6H_2_O, FeCl_2_·4H_2_O and methylene blue were purchased from Beijing Chemical Reagent Plant and (C_4_H_9_)_4_NOH was obtained from Sinopharm Chemical Reagent Co., Ltd.

### Preparation

The GO nanosheets were fabricated by Hummers’ method^43^. Titania nanosheets were prepared by exfoliating the layered titanate as reported previously^44^. Fe_3_O_4_ was synthesized as follows. An aqueous solution (50 mL) containing FeCl_3_·6H_2_O (0.4953 g) and FeSO_4_·7H_2_O (0.2547 g) was added into a vessel with water ultrasonic, stirred quickly and kept at the temperature of 65 °C. NH_3_·H_2_O was slowly added till pH reached 10. Then the mixture was subjected to ultrasonication and stirred for 1 h.

The particles were collected by a magnet and washed three times with distilled water. The final samples were dried in a vacuum at 80 °C for 8 h. 10 mL mixture of GO (0.1 mg/mL) and titania nanosheet (0.08 mg/mL) was stirred for 1 h, and the volume ratio of GO solution and titania nanosheet solution was 5%. Then 0.1 mg Fe_3_O_4_ particles was added under stirring. At last 5 mL PDDA (20 g/L, pH = 9) aqueous solution was added to the mixture dropwise with stirring for 1 hour, and composite particles were flocculated out of the aqueous dispersions. The composite was subsequently calcinated at 300 °C for 1 h.

### Characterization

The morphology and elemental distribution analysis were characterized on a scanning electron microscope (SEM, SU1510, Hitachi Ltd., Tokyo, Japan) equipped with EDS. The thickness of GO and titania nanosheet was determined by atomic force microscopy (AFM, Dimension 3100, Veeco, USA). The zeta potential was tested using Zetasizer Nano ZS90. X-ray photoelectron spectroscopy (XPS) was conducted on an ESCALAB 250 photoelectron spectrometer (Thermo Fisher Scientific) using a Kratos Axis Ultra system with a monochromatized Al Kα radiation at 1486.6 eV as the X-ray source. Flourier-transform infrared (FT-IR) spectra was recorded from 400–4000 cm^−1^ on the 100 FTIR spectrometer (Peikin Elmer., USA). The diffuse reflectance spectra (DRS) was measured from 200–2000 nm using a PerkinElmer Lambda 35 UV-vis spectrophotometer which was equipped with integrating spheres, and BaSO_4_ was used as a reference. Electrochemical and photoelectrochemical measurements were measured on a CHI660C electrochemical workstation in a three-electrode system. The as-prepared sample was the working electrode, Pt wire was the counter electrode, and platinum-saturated calomel electrode (SCE) was the reference electrode. The light source was a 300 W Xenon lamp equipped with a UV light filter. The photoresponses of photocatalysts were measured at 0.0 V. Electrochemical impedance spectra (EIS) was characterized at 0.0 V. Inductively-coupled plasmas mass spectrometry (ICPMS, Perkin Elmer Optima 8300 Series) was used to determine the concentration of the titania nanosheet. Electrochemical characterization was conducted using an electrochemical workstation (CHI660 C, ShanghaiChenhua) with a three-electrode cell system. The total organic carbon (TOC) was measured on a total organic carbon analyzer (Shimadzu, TOC-L-CPH). GC-MS (Teacel130, ISQQD) was used to determine the residues in the degraded water. The electrical conductivity of the electrolyte was determined by a conductivity meter (DDSJ-318).

### Photocatalytic activity experiments

The photocatalytic activities of the samples were evaluated by degradation of Methylene blue (MB) in solution under visible light. A Xe light (500 W xenon lamp, λ > 420 nm) was used as a light source. 10 mg photocatalytic sample was added in a 40 mL MB solution (3 × 10^−5^ M) under magnetic stirring. The solution was stirred in the dark for 1 hour to reach the adsorption equilibrium before illumination. At fixed time intervals, 3 mL of the mixing suspension was taken out and then was separated by placing a magnet beside the reaction vial followed by pouring out the solution. The solution was measured by UV-vis spectra and the intensity of 665 nm was displayed in the diagram.

### Active species trapping test

In experiments to determine the active species in the photocatalytic experiment, 1 mM EDTA-2Na, 1 mM p-benzoquinone (BQ) and 1 mM isopropanol (IPA) were used to capture holes (h^+^), superoxide radicals (•O^2−^) and hydroxyl radicals (•OH), respectively. These active species scavengers were added individually during the photocatalytic reaction. The method of active species trapping experiment was the same as photocatalytic activity experiments.

### Electrochemical reactions

Cyclic voltammetry (CV) and linear sweep voltammograms (LSV) was used to evaluate the ORR performance. The catalyst samples were loaded on the glass carbon electrode as the working electrode, a Pt wire was employed as the counter electrode and saturated calomel electrodes as the reference electrode. The working electrode was prepared by mixing 5 mg samples, 5 mg carbon black and 10 μL Nafion solution. The 0.1 M KOH electrolyte was saturated by constant O_2_ purging. The LSV measurements were scanned at 1600 rpm with a 5 mV•s^−1^ scan rate. The electrolyte prepared using the photocatalytically treated water was prepared by mixing the treated water and deionized water at 1:1 ratio, and into the mixture added the corresponding amount of KOH.

## Results and Discussions

Figure 1 illustrates the synthetic procedures of the magnetic composite photocatalyst. The titania nanosheets were obtained by exfoliation from the layered titanate crystal in a tetrabutylammonium hydroxide solution^44^. The obtained nanosheets were most single or double layered titania, with a thickness within 1–2 nm (Fig. S1). The iron element was included into the catalyst by introducing the magnetic Fe_3_O_4_ nanoparticles^45^. Graphene oxide (GO) nanosheet was prepared using Hummers methods and the characterizations were displayed in Fig. S2 ^43^. The exfoliated titania sheets (zeta potential −48.5 mV), GO(zeta potential −47.5 mV), and magnetic iron oxide (zeta potential −36.4 mV) were all negatively charged. Upon adding a positively charged PDDA, the four precursor component of the magnetic catalyst flocculated out of the solution because of electrostatic attractions. Afterward, the precipitation was annealed at 300 °C for 1 h in air to obtain the composite catalyst.

**Figure 2:**
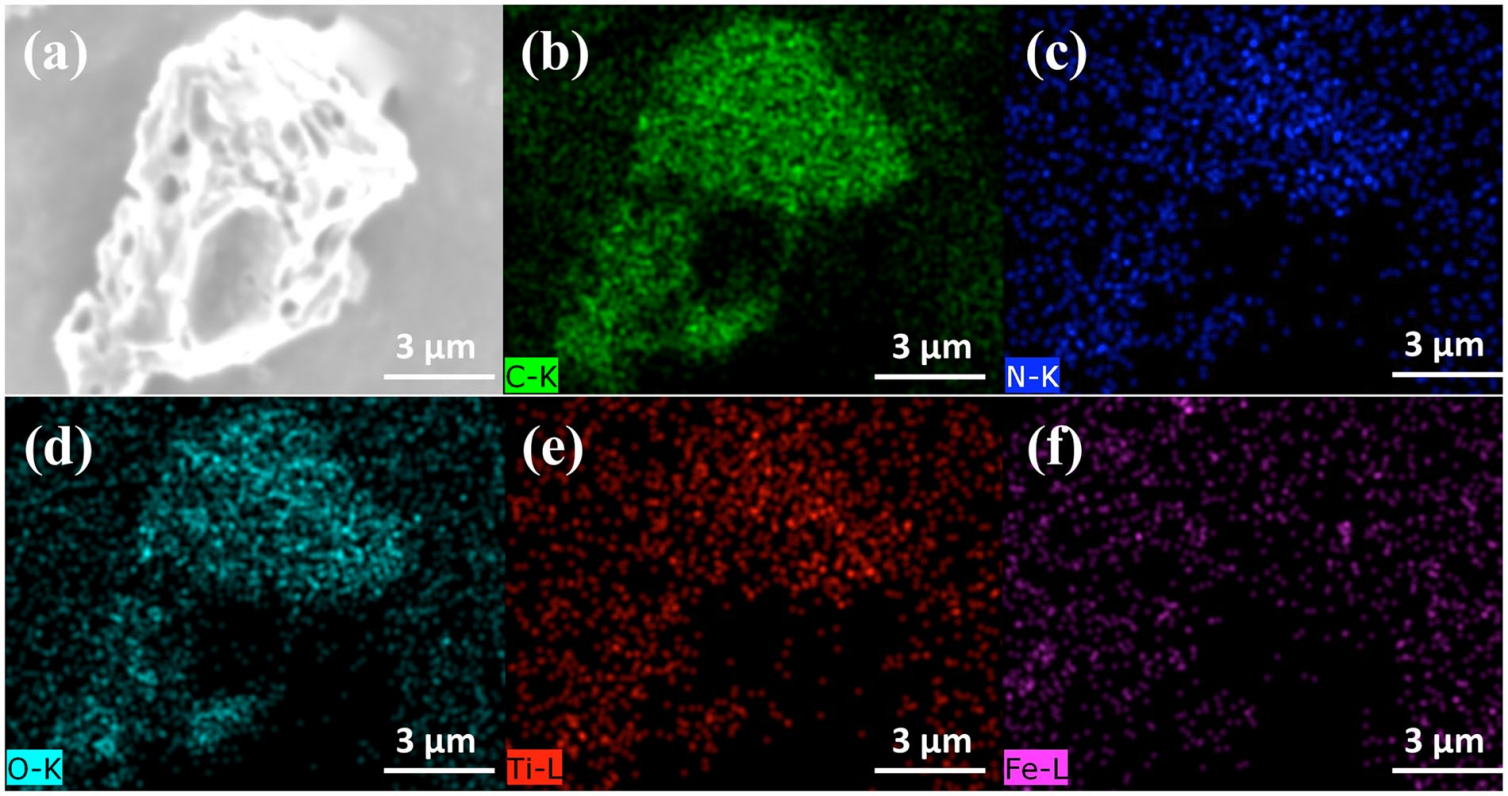
SEM image (**a**) and the mapping of the (**b**) carbon, (**c**) nitrogen, (**d**) oxygen, (**e**) titanium, and (**f**) iron elements of the magnetic photocatalysts.

The morphology and elemental distributions of the magnetic photocatalyst were investigated by scanning electron microscopy (SEM) and EDS elemental mapping as shown in Fig. 2. The SEM images indicated that the magnetic photocatalyst was particles with lateral sizes in the micrometre-scale. Pores of tens to hundreds of nanometers were observed on the particles. Elemental mapping showed that C, O, N, Ti, Fe elements distributed homogeneously throughout the particle surfaces, and the dispersion areas of these elements overlapped, indicating that the components in the catalyst distributed evenly. The elemental content fractions were shown in Table S1.

**Figure 3:**
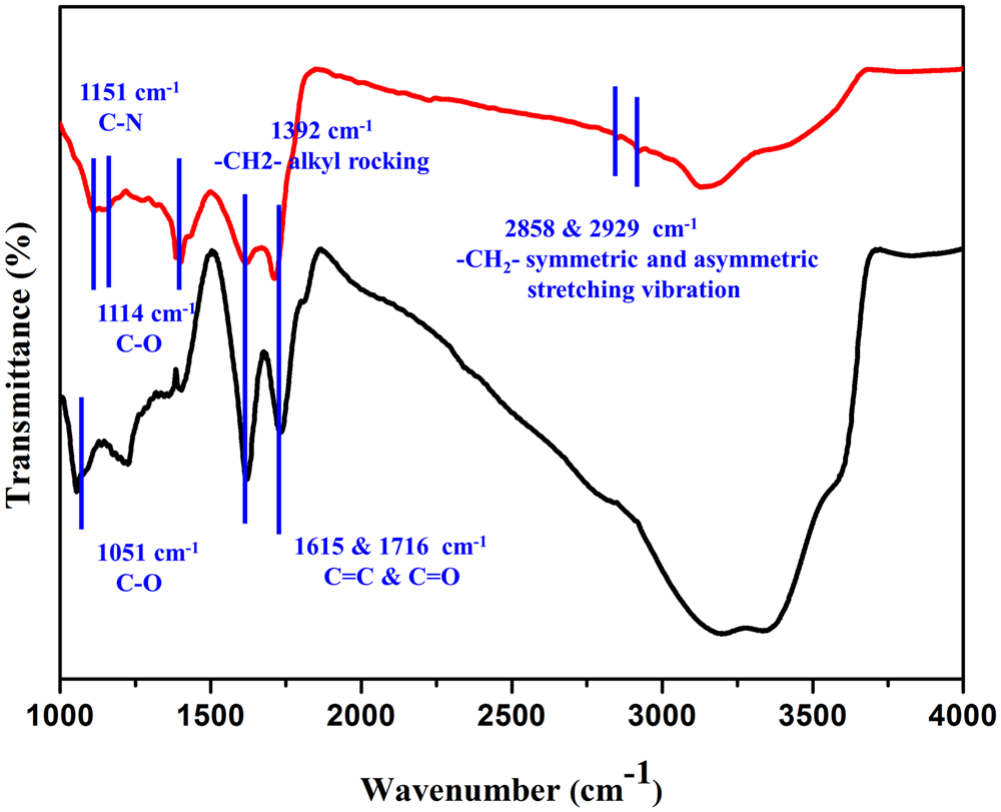
FTIR spectra of the composite photocatalyst (red line) and GO (black line).

The components and functional groups in the composite catalyst were further studied using FTIR spectra, shown in Fig. 3. FTIR results verified the changes of GO and partially carbonated PDDA in the composite catalyst. Vibrations at 2929 cm^−1^, 2858 cm^−1^, 1392 cm^−1^ and 1151 cm^−1^ were attributed to the -CH_2_− asymmetric stretching vibration, the -CH_2_− symmetric stretching vibration, the -CH_2_− alkyl rocking and the C-N stretching vibrations, respectively. These vibrational bands indicated that the polymeric component PDDA was only partially carbonized after calcination, and the organic component of PDDA was still preserved. These organic functional groups played important roles in boosting the catalytic power as studied previously^46^. In Fig. 3b, Vibrational bands at 1716 cm^−1^, 1615 cm^−1^ and 1114 cm^−1^ were assigned to GO^47^. FTIR characterization was consistent with TGA results for PDDA and GO, that during calcination at 300 °C, GO was mostly reduced to rGO, and PDDA was partially carbonized as shown in Figure S3 ^46^.

**Figure 4:**
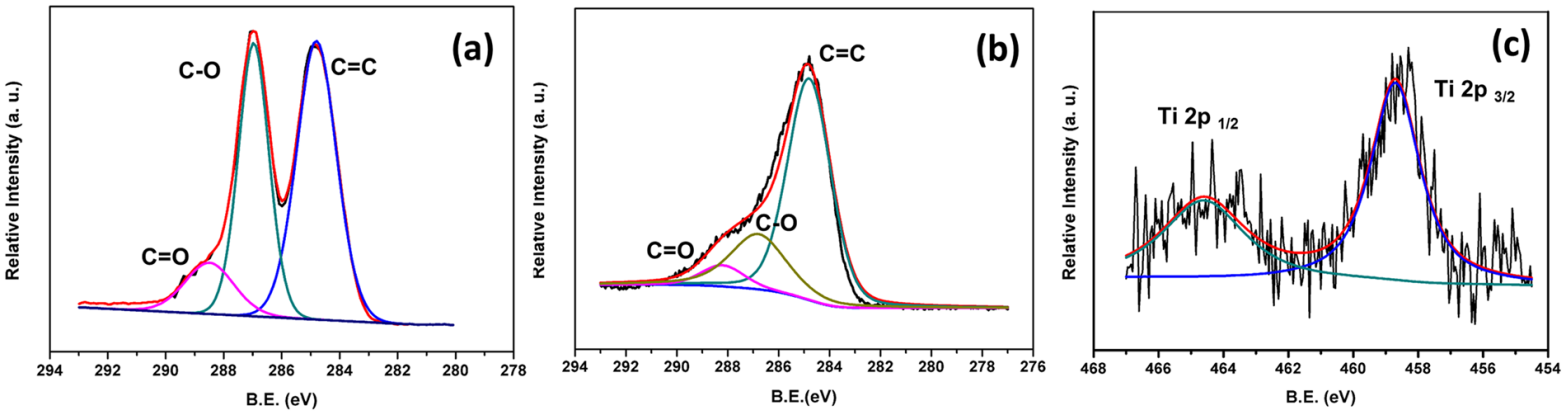
C 1 s XPS spectra of (**a**) Graphene oxide and (**b**) the composite photocatalyst (**c**) Ti 2p XPS spectra of the composite photocatalyst.

The argument that GO was substantially reduced during calcination was further supported by XPS in Fig. 4. The C 1 s spectra of GO and the composite catalyst (with 5% GO in the precursor mixture) were studied and compared. The three peaks at 284.8 eV (sp2 hybridized carbon in graphene oxide, C=C), 286.8 eV (carbon in hydroxyl and epoxide group, C-O), and 288.2 eV (carbonyl carbon, C=O)^48–50^ displayed clearly different relative intensities in the spectra of GO and in the catalyst. In GO samples, the peak intensities for C-O and C=C were almost identical, while in the catalyst the peak intensities for C-O and C=O was remarkably weakened (to around 30%) relative to the intensity for C=C. This clear difference in relative peak intensities in GO and the catalyst indicate that GO was substantially reduced in the composite catalyst. The relative intensities of C-O, C=O, and C=C peaks instructed the reduction status of GO in the catalyst because only GO or rGO presented these groups, and other component in the composite catalyst -PDDA, titania nanosheets, magnetic particles- didn’t carry these groups. In addition, the band located at binding energies of 464.6 eV and 458.7 eV in the composite catalyst were assigned to Ti-O bonds in the titania nanosheets^51^.

**Figure 5:**
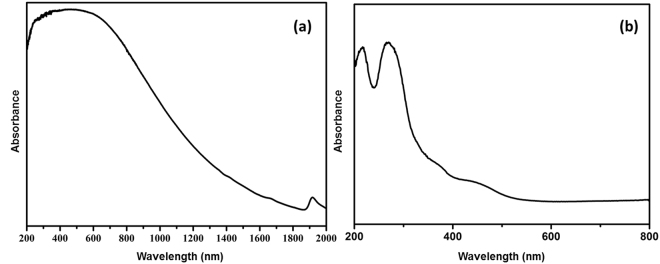
UV-Vis DRS of (**a**) the composite photocatalyst and (**b**) the titania nanosheet.

The composite catalyst displayed extremely broad light absorbance properties throughout UV, Vis, and NIR range. UV-vis diffuse reflectance spectra (DRS) of the magnetic composite catalyst displayed strong absorbance between 200 and 750 nm, covering almost entire UV-vis ranges. Beyond 750 nm, the absorbance gradually decreased and reached a minimum at as far as 1800 nm. Thus not only in the UV-vis range, but also in the NIR range, the composite catalyst absorbed light effectively. This large absorbance range made our composite photocatalyst quite unique as photocatalysts. The partially carbonized PDDA was believed to have contributed mostly to this unique absorbance characteristics and rGO assisted in elevation of the absorbance throughout UV-vis range^52–54^. The enhanced and extended absorbance spectra was greatly improved compared with the absorbance of titania nanosheets, which absorbed only in the UV range through 200–400 nm (Fig. 5). Interestingly, the magnetic particles not only contributed magnetic property to the composite particles, but also enhanced light absorbance in the NIR range (Figure S4). The band gap energy (Eg) of the magnetic composite particles was calculated as 0.70 eV (according to the formula Eg = 1240/λ (eV)) as opposed to 0.63 eV for the control particles prepared in the absence of the magnetic particles^55^. The narrow band gap of the iron oxide particles renders it effective light absorbance in NIR range and contributed to the NIR light absorbance of the composites^56^.

**Figure 6:**
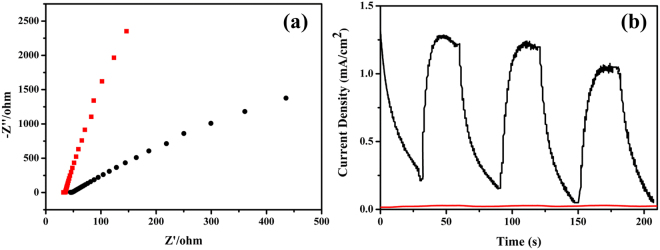
(**a**) Electrochemical impedance spectra of the composite photocatalyst (black dot line) and the pure titania nanosheets (red dot line). (**b**) Photocurrent of the composite photocatalyst (black line) and the pure titania nanosheets (red line).

Electrochemical impedance spectra (EIS) analysis (Fig. 6a) indicated that the composite catalyst presented improved electron-hole separation efficiency compared with exfoliated titania nanosheets. In Nyquist plot of the composite catalyst, the radius of the semi-sphere in the low resistance range was smaller than in the plot of the titania nanosheets, indicating that the electron-hole separating rate was larger for the composite catalyst than titania nanosheets. The enhanced electron-hole separation rate would be beneficial in obtaining superior photocatalytic power, because effective electron-hole separation generates active site to take part in reactions.

The enhanced light absorbance capabilities and a larger electron-hole separation rate of the composite catalyst in turn resulted in heightened photocurrent generations upon visible light irradiation, as shown in Fig. 6b. The intense photocurrent was generated immediately and quickly reached the maximum value upon visible light irradiation, and diminished rapidly once the light was removed. The repeated cycles of photocurrent generation and diminish cycles indicated effective light responsive nature of the composite photocatalyst. The superior light responsive properties were attributed to the existence of graphene, partially carbonized PDDA, and the magnetic particles. These three components facilitated electron transfer because of electron-withdrawing abilities (PDDA), electric conductive properties (rGO), and an appropriate band position (magnetic particles), and thus assisted in obtaining strong photocurrent intensities^46,53,57^. In clear comparison, the exfoliated titania nanosheets, which barely absorbed visible light, hardly generate any photocurrent under visible light irradiation. In addition to the heightened light absorbance and electron-hole separation properties, the composite particles presented magnetic responsive capabilities. A saturated magnetization value of 0.96 emu/g was obtained for the composite particles.

Photoluminescence (PL) spectrum was employed to investigate the separation efficiency of photo-generated electrons and holes. As shown in Figure S5, the PL intensity of composite was lower than that of the titania nanosheet, indicating that the separation of photo-generated electron and holes in the composites were more efficient than those in pure titania nanosheet. The results were consistent with electrochemical impedance spectra (EIS) analysis.

**Figure 7:**
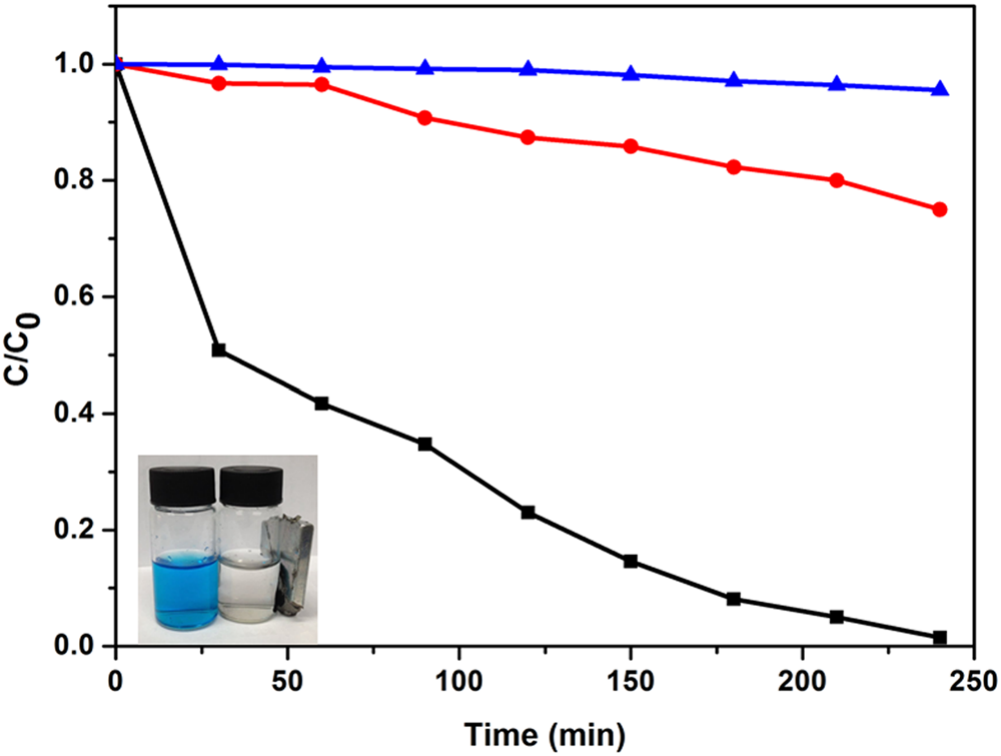
Photocatalytic degradation of MB using the pristine titania nanosheet (blue line), P25 (red line) and the composite photocatalyst (black line). The insert is an optical image indicating that the composite photocatalyst can be recycled by a magnet.

**Figure 8:**
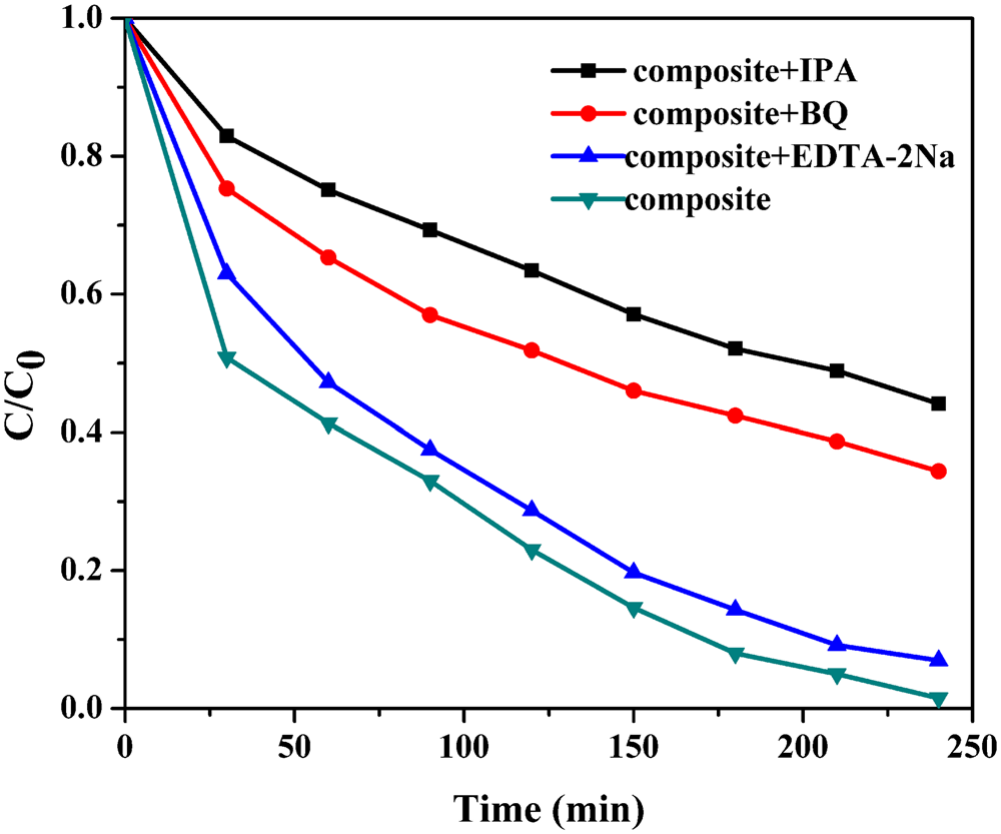
Photocatalytic degradation of MB in the presence of different scavengers.

**Figure 9:**
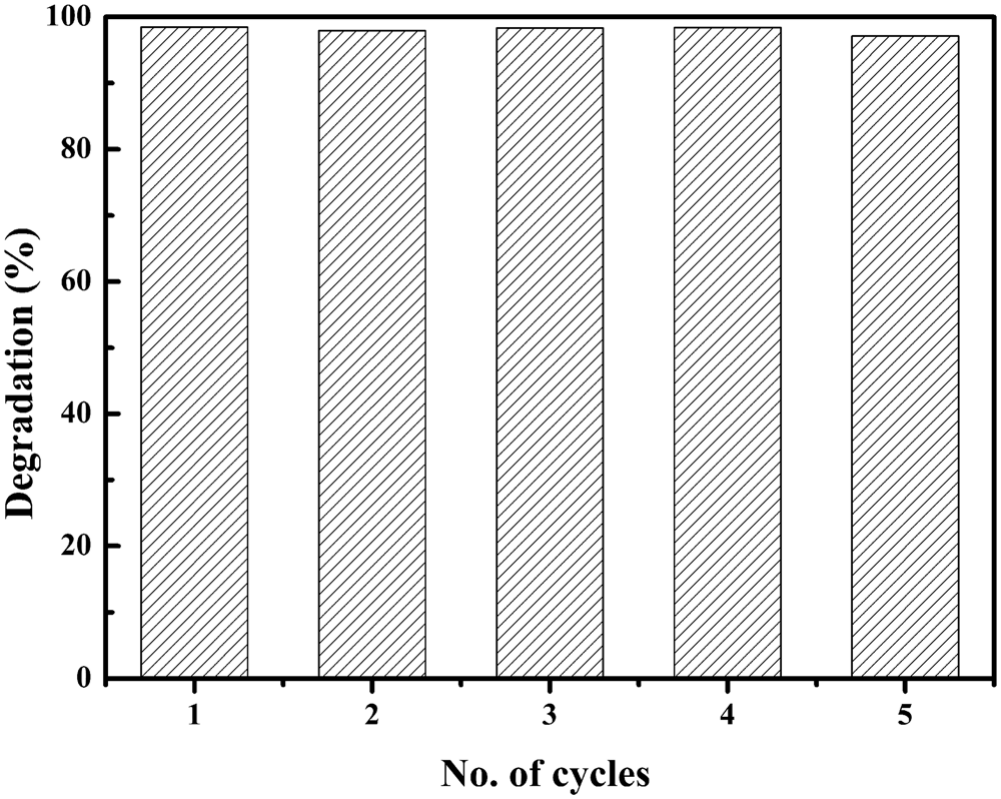
The cycled photocatalytic degradation of MB by the composite photocatalyst.

The physical-chemical characterizations of the composite particles strongly suggested that these particles would present advantageous photocatalytic properties. The photocatalytic power of our composite particles was demonstrated using methylene blue (MB) catalytic degradation reaction. Commercial titania catalyst P25 and pristine exfoliated titania nanosheets were used as a reference for catalytic performance evaluations. Previous studies indicated that GO fractions in the composite particles strongly influenced the catalytic performance. Serial studies here indicated that GO fraction of 5% gave the optimized catalytic power (Figure S6). As shown in Fig. 7, the degradation efficiency of the composite magnetic photocatalysts (GO fraction 5%) clearly outperformed P25 and pristine titania nanosheets. When 99% MB absorbance was removed by our composite catalyst at 215 min, only 25% MB absorbance was removed by P25 and the pristine titania sheets hardly removed any MB absorbance. After the photocatalytic reaction, the composite catalyst could be removed from the mixture (Fig. 7) and recycled taking advantage of its magnetic properties. In order to further investigate the photocatalytic mechanism, the trapping method was carried out to identify the catalytic active species. EDTA-2Na, p-benzoquinone (BQ) and isopropanol (IPA) were introduced to act as the scavengers of holes (h^+^) superoxide radicals (•O^2−^) and hydroxyl radicals (•OH), respectively. As shown in Fig. 8, the degradation performance of MB was severely impacted when BQ and IPA were added into the system. These results indicated that •O^2−^ and •OH were the main active species under the visible light irradiation. And the photocatalytic activity of composite was almost unchanged by the addition of EDTA-2Na, indicating the holes were not the oxidative species. Based on the above information, the proposed mechanism of the photocatalytic process was that under visible light irradiation, the electron was exited from carbonated PDDA or GO, and then injected to the surface of titania nanosheet to form radicals (•O^2−^ and •OH)^46,53^, as shown in Figure S9. The photocatalyst was further tested for its recyclability. After 5 cycles of applications, negligible performance deterioration (less than 2%) was observed for our composite catalyst (Fig. 9). The stable performance in addition to the effective catalytic properties of our composite magnetic catalysts demonstrated their superior catalytic performance.

The photocatalytic decomposition was monitored by UV-vis spectra. After 250 min, the absorbance of the residue solution reached zero, indicated a 100% decomposition of MB. This performance was comparable to best catalysts in MB decompositions^18^. We stopped the reaction when the absorbance reached zero because the absorbance was frequently used as a viable indicator of the completion of the decomposition reaction. However, when we proceeded to test TOC of the residue solution, and obtained a high value of 64.7%, indicating the existence of organic residues in the treated water. The high TOC value highlighted the necessity to further study the possible applications of the degraded water, considering its possible toxicity. Propelled by this consideration, we proceeded to explore the application of the degraded water as a component of the ORR electrolyte.

**Figure 10:**
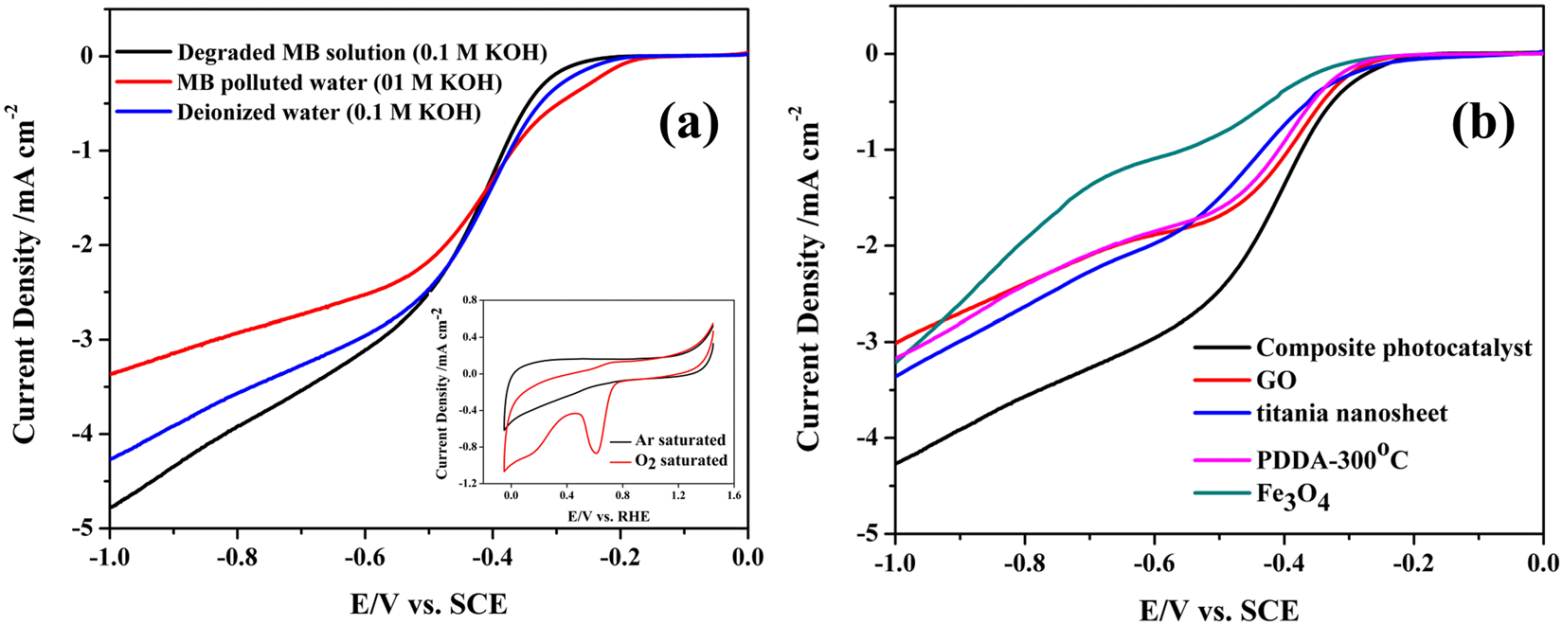
Electrochemical characterization in a solution of O_2_-saturated KOH (0.1 M) at a rotation speed of 1600 rpm. (**a**) LSV of composites in the electrolyte prepared using the MB-polluted water (0.1 M KOH, red line), in a regular electrolyte prepared using deionized water (0.1 M KOH, blue line) and in the electrolyte prepared using the photocatalytically degraded MB dye solution (black line). The insert image is CV curves of the composite catalyst in Ar- and O_2_− saturated KOH solution (0.1 M, prepared using deionized water) at a scan rate of 50 mV s^−1^. (**b**) LSV curves of the composite catalyst and a variety of reference samples (including GO, titania nanosheets, PDDA calcinated at 300 °C, and Fe_3_O_4_ nanoparticles) at the same test condition.

In order to demonstrate the possible application of the photo-catalytically treated water, we carried out electrochemical reductive reactions in a solution prepared using the treated water. We chose the electrochemical reduction reaction to demonstrate the application of the treated water because a variety of redox active species were believed to be able to contribute to the reductive electric current^58^ and the possible chemical residues in a photo-catalytically treated water should be allowed in such applications. The prepared composite catalyst was used as the cathode in the reductive reactions. The prepared catalyst was expected to possess ORR catalytic activity because of the existence of Iron oxide. Titania and the carbonized polymer as well as graphene should facilitate the electronic transfer and further enhance the ORR activities^48,59,60^. Although most studies regarding ORR focused on the development of catalyst, the component of the electrolyte is always a practical issue to be considered in experiments^61,62^. The influences of the electrolyte on Pt/C catalyst have been well documented, and were also studied for Fe-based catalyst^18^. These studies found that the ions in the electrolyte may decrease the ORR performance by blocking the active sites of the catalysts. Thus whether the degraded water can be used as the ORR electrolyte would be a practical issue to be verified. Compared with the solution saturated with Ar which presented only capacitance behavior, the oxygen-saturated electrolyte gave a clear reductive peak and remarkably higher current densities in CVs (Fig. 1 inset), indicating that the composite catalyst performed as the catalyst cathode in ORR reaction. In the electrolyte saturated with oxygen, linear sweep voltammograms (LSV) were used to study the electrochemical activities of the electrolyte, which should involve the electrochemical reduction of organic compounds and ORR activity. As shown in Fig. 10(a) the onset voltage of LSV in the MB-polluted electrolyte was higher than in the regular electrolyte prepared using the deionized water, but the value of the current density in the MB-polluted electrolyte was remarkably smaller (−3.4 mA·cm^−2^) than in the regular electrolyte (−4.2 mA·cm^−2^) at −1.0 v. The reductive current from the regular electrolyte should be provided by the ORRs. A higher onset voltage and a lower current density at −1.0 v in the MB-polluted electrolyte indicated that the reductive current was contributed by not only ORR but also the reduction of other compounds, possibly the organic dye MB^58^. However, at potentials negative than −0.55 v, the absolute value of the current density was remarkably smaller in the MB-polluted electrolyte than in the regular electrolyte, possibly due to the passivation of the catalyst electrode by the organic compounds^63^. These results indicate that although the MB-polluted water provided larger types of reductive active species for the electrochemical reaction, the complex solution at the same time passivated the catalyst electrode, resulting in a compromised electrode performance. After photocatalytic decomposition, the treated water was used to prepare the electrolyte and in clear comparison to the previously mentioned electrochemical reactions in the MB-polluted electrolyte, the absolute value of the current density here was even larger than the one in the regular electrolyte. The possible reason for the increase of the current density might result from the existence of extra reactive active species after MB decomposition. Benzothiazole, azure A and maleic acid were detected in the treated water by GC-MS. And in the previous study, inorganic ion NO_3_^−^, Cl^−^ and SO_4_^2−^ also were found in degraded MB solution^61^. These organic species and ions can enhance the conductivity of electrolyte^64^. The electrical conductivity of the electrolyte containing photocatalytic degraded MB solution (22.1 mS/cm) was larger than that containing MB solution (21.7 mS/cm).

Here we demonstrated the electrochemical catalytic effect of the composite catalyst using a series of reference materials. Compared with GO, carbonized PDDA, the titania nanosheets, and iron oxide nanoparticles, the composite catalyst gave the highest catalytic ORR performance. Shown in Fig. 10b, in a standard ORR condition, the composite catalyst afforded a more positive onset potential (−0.2 V vs. SCE) and largest current density (−4.25 mA·cm^−2^ at −1.0 V). In comparison, the current density obtained from all other types of cathodes were more positive than −3.5 mA·cm^−2^. In order to understand the exact role of each component of the quarternary composite in ORR reaction, we conducted a series of control experiments by preparation of four types of ternary catalysts by excluding one single component in each sample. As shown in Figure S8, the current density and onset potential of the ternary samples with PDDA (TNs/GO/PDDA, GO/Fe_3_O_4_/PDDA, TNs/Fe_3_O_4_/PDDA) were higher and more positive than TNs/GO/Fe_3_O_4_, indicating that PDDA played an important role in this ORR reaction. Here, in addition to the function of forming nitrogen-doped carbon after calcination, PDDA not only facilitate interactions with the electrolyte due to its hydrophilic property, but also facilitated O_2_ adsorption because of its electron-withdrawing property^64,65^. TNs/GO/PDDA on account of combined GO and titania nanosheet (two kinds of two dimensional nanosheet) possessed the most positive onset potential (−0.05 V vs. SCE), due to the larger contact area, better dispersity and more exposed activated sites^66^. Compared with TNs/Fe_3_O_4_/PDDA and GO/Fe_3_O_4_/PDDA, GO/Fe_3_O_4_/PDDA with similar onset potential emerged larger current density, as a result of high electrical conductivity of GO. Fe_3_O_4_ also contributed to the overall current density because Fe (II) was studied as active O_2_ adsorption site in ORR catalysts^18^. The ternary catalyst left out TNs possessed a current density approaching that of the quaternary catalyst, indicating that TNs played a minimum role in enhancing ORR current density. But TNs should enhance the stability of the composite catalyst and were indispensable in decomposing MB^3,21,22^. In summary, Due to the synergistic effect of titania nanosheet, GO, PDDA and Fe_3_O_4_, the composite had a more positive onset potential (−0.2 V vs. SCE) and largest current density (−4.25 mA·cm^−2^ at −1.0 V).

## Conclusions

We try to demonstrate a concept of multiple and cyclic application of materials and resources in environmentally relevant catalyst reactions. The concept was enabled by a catalytically bifunctional magnetic catalyst that can not only degrade organic dyes in solutions but is also electrochemically active and can catalyst ORRs. The MB solution after photocatalytic decomposition can be used to prepare electrolyte for ORRs. The cyclic use of the photocatalytically cleaned water and the bifunctional use of the composite catalyst should aid in environmental remedy. The catalyst was prepared using a facile “flocculation followed by calcination” strategy, and its multiple uses depended on the delicate choice of the components, each of which was indispensable in its catalytic activity, but each component played different roles in the photochemical, magnetic recycling, and electrochemical processes. Though the overall performance and the performance evaluation method should be further studied and developed for such a concept, we expect this report inspire the researches in the design of multiple application of a single material and provide a novel idea in environmental remedy.

## Electronic supplementary material

supplementary information
