# Supplementary material for: Constructing the magnetic bifunctional graphene/titania nanosheet-based composite photocatalysts for enhanced visible-light photodegradation of MB and electrochemical ORR from polluted water
Source: Sci Rep. 2017 Sep 25;7:12296. doi: 10.1038/s41598-017-12504-2 (PMC5612995; doi:10.1038/s41598-017-12504-2)
Supplement: Supplementary file 1 — supplementary information [file 41598_2017_12504_MOESM1_ESM.doc]

Supplementary Information

**Constructing the magnetic bifunctional graphene/titania nanosheet-based composite photocatalysts for enhanced visible-light photodegradation of MB and electrochemical ORR from polluted water**

Qian Zhang^1,2^, Yihe Zhang^1^*, Zilin Meng^1,2^, Wangshu Tong^1^, Xuelian Yu ^1^*, Qi An^1^*

^1^ Beijing Key Laboratory of Materials Utilization of Nonmetallic Minerals and Solid Wastes, National Laboratory of Mineral Materials, School of Materials Science and Technology, China University of Geosciences, Beijing, 100083

^2^ School of Resources and Environmental Engineering, Shandong University of Technology, Zibo, 255049

Email: zyh@cugb.edu.cn; xlyu@cugb.edu.cn; an@cugb.edu.cn.


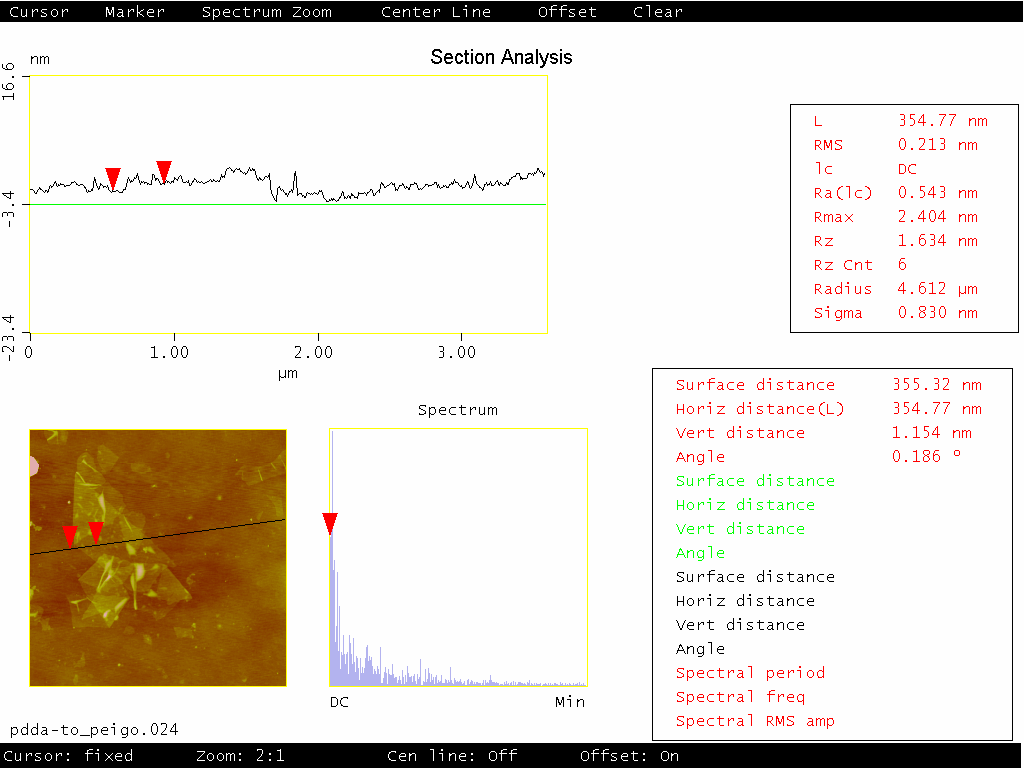


**1 μm**


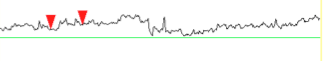


**1.2nm**

Fig S1. AFM image and the thickness analysis of the exfoliated titania nanosheet.


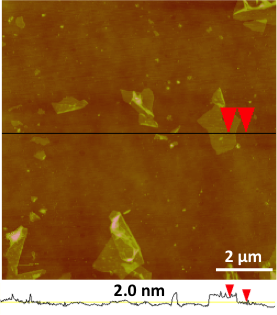


Fig S2. AFM image and the thickness analysis of the graphene oxide.


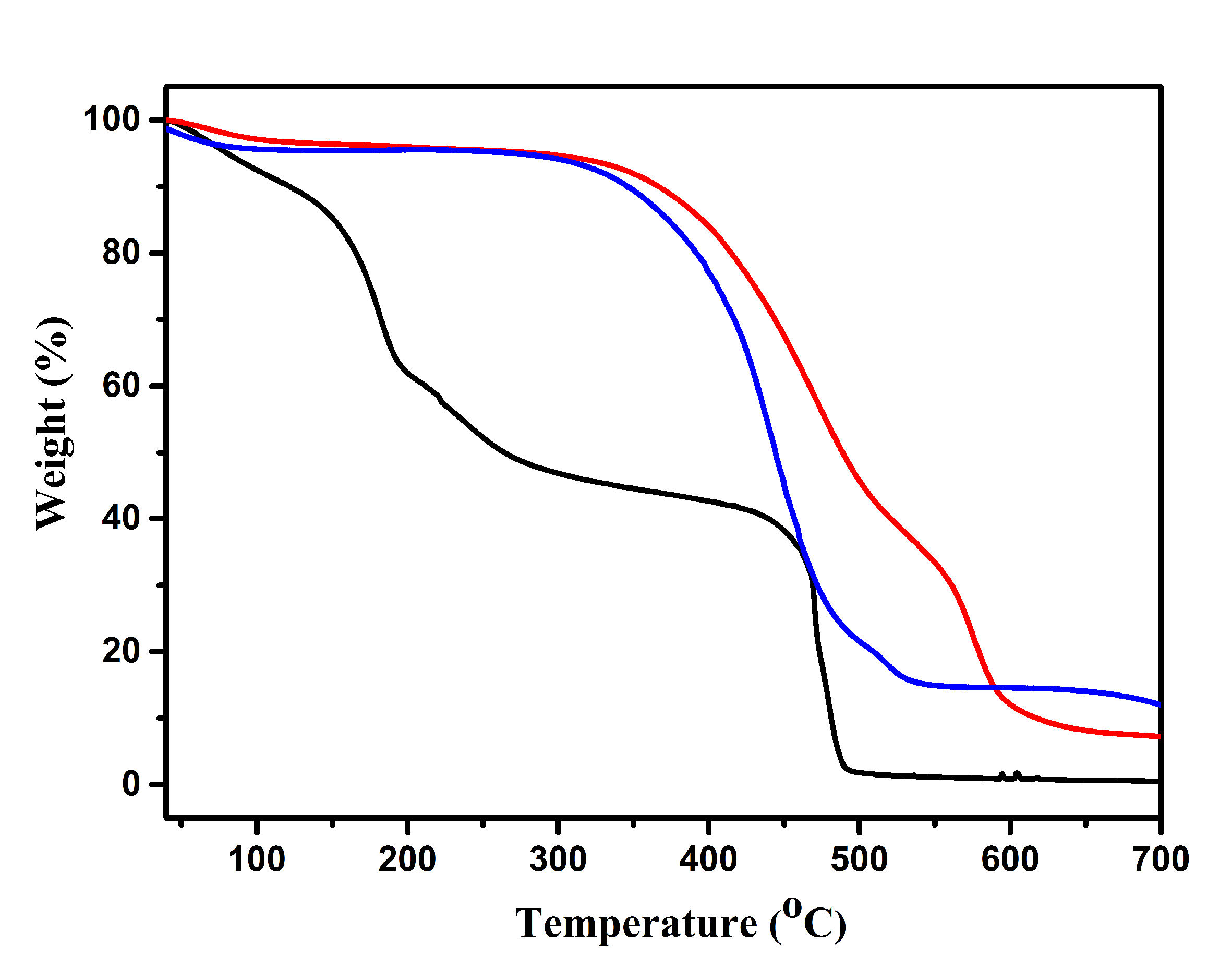


Figure S3. TGA curves of composite photocatalyst (blue line), GO (black line) and PDDA (red line).


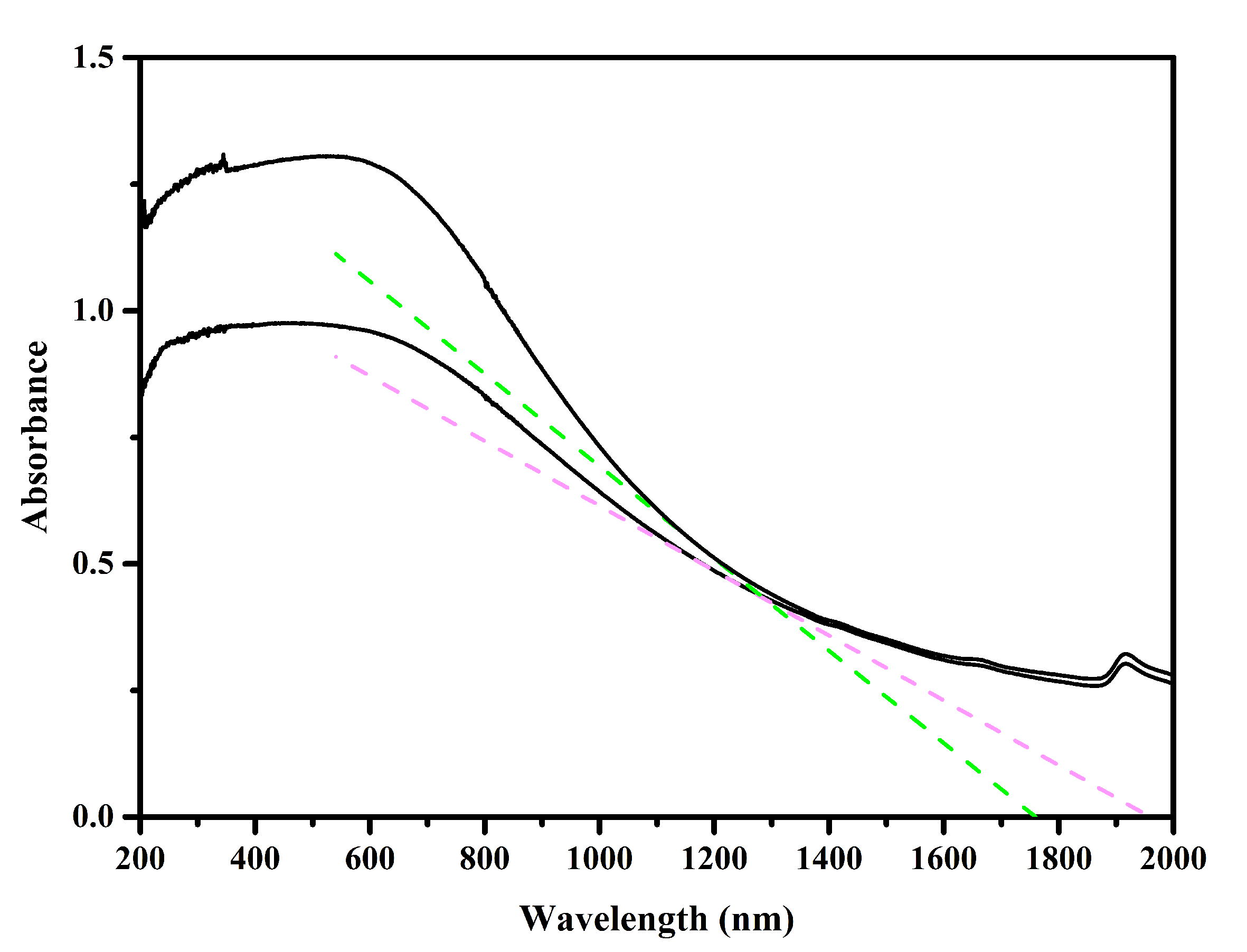


Figure S4. UV-Vis diffuse reflectance spectra of (a) composite photocatalyst without magnetic particle (b) magnetic photocatalyst.


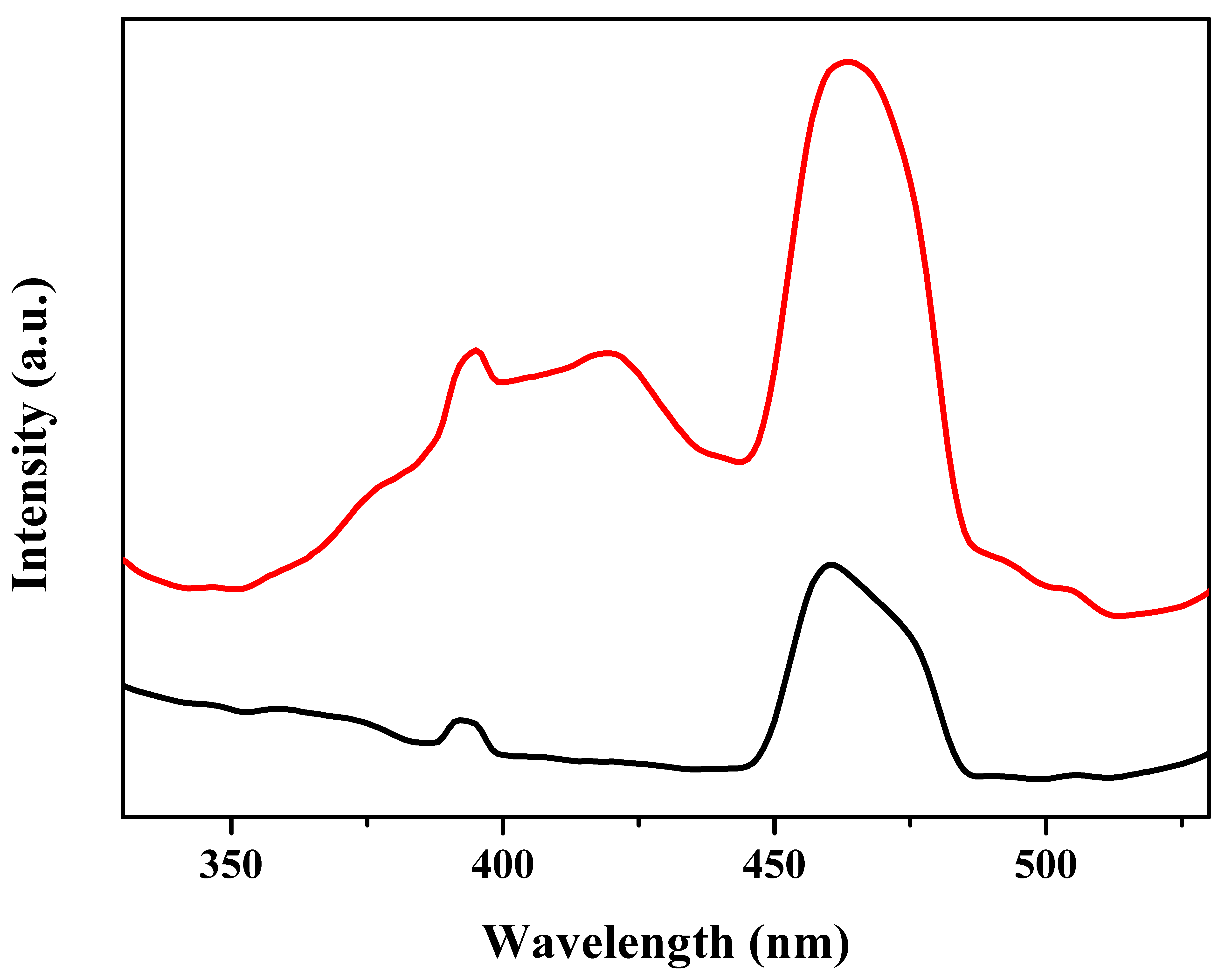


Figure S5. Photoluminescence (PL) spectra of the titania nanosheet (red line) and the composite photocatalyst (black line).


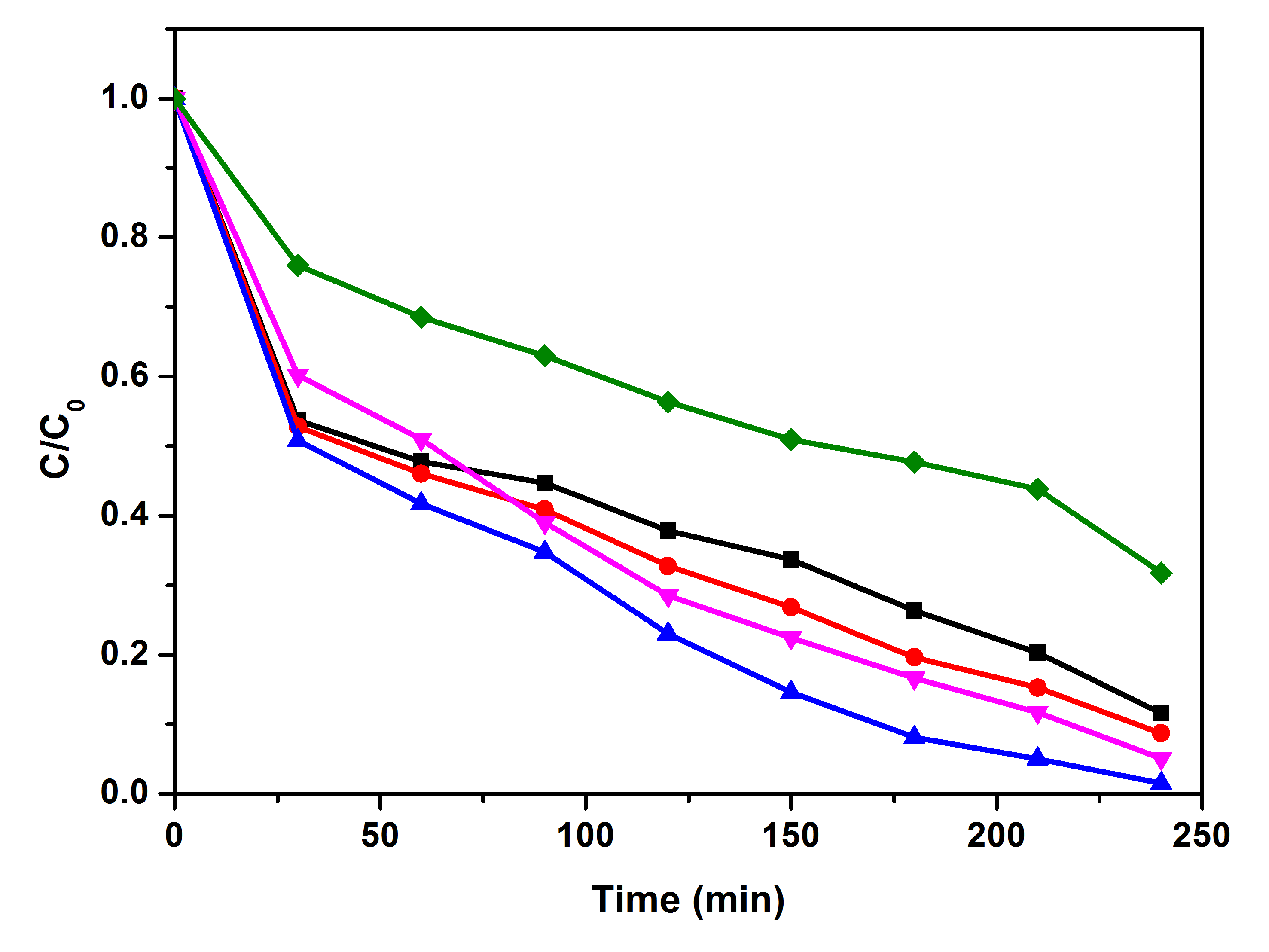

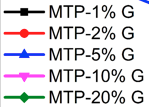


Fig S6. Degradation efficiency of the composite magnetic photocatalysts with different GO contents.


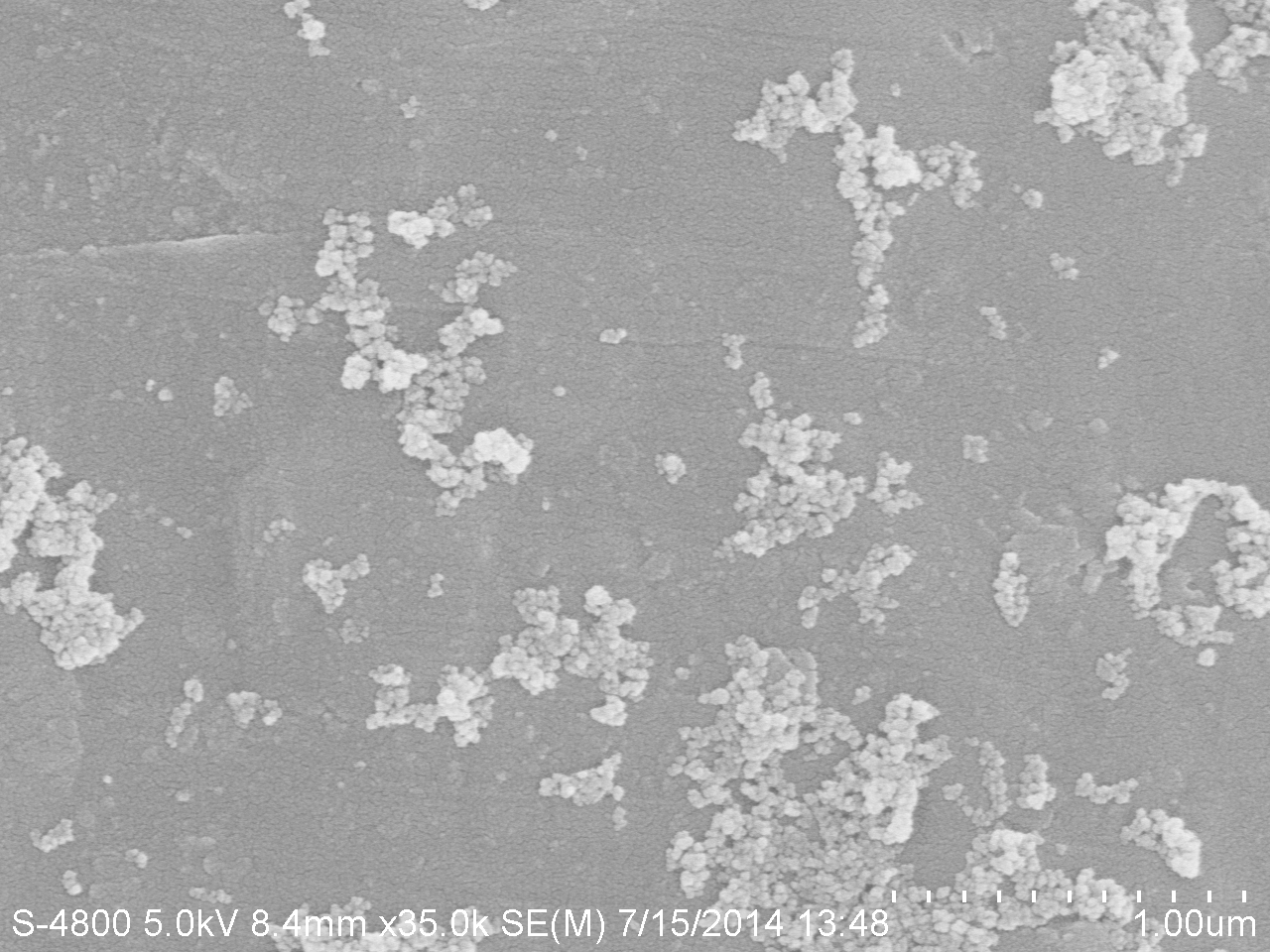


Figure S7. SEM image for Fe_3_O_4_ nanoparticle.


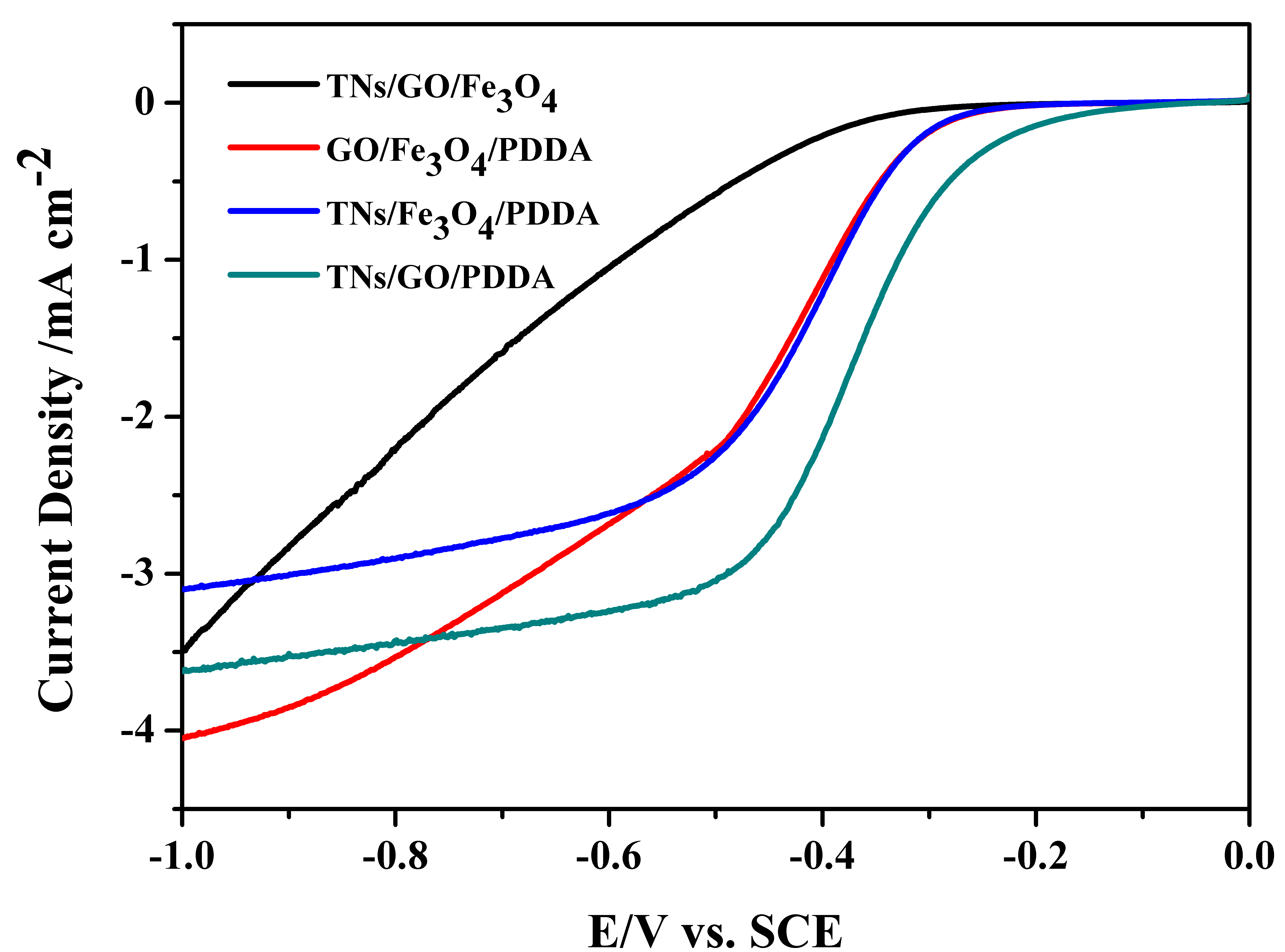


Figure S8. LSV curves of reference samples of ternary catalysts.


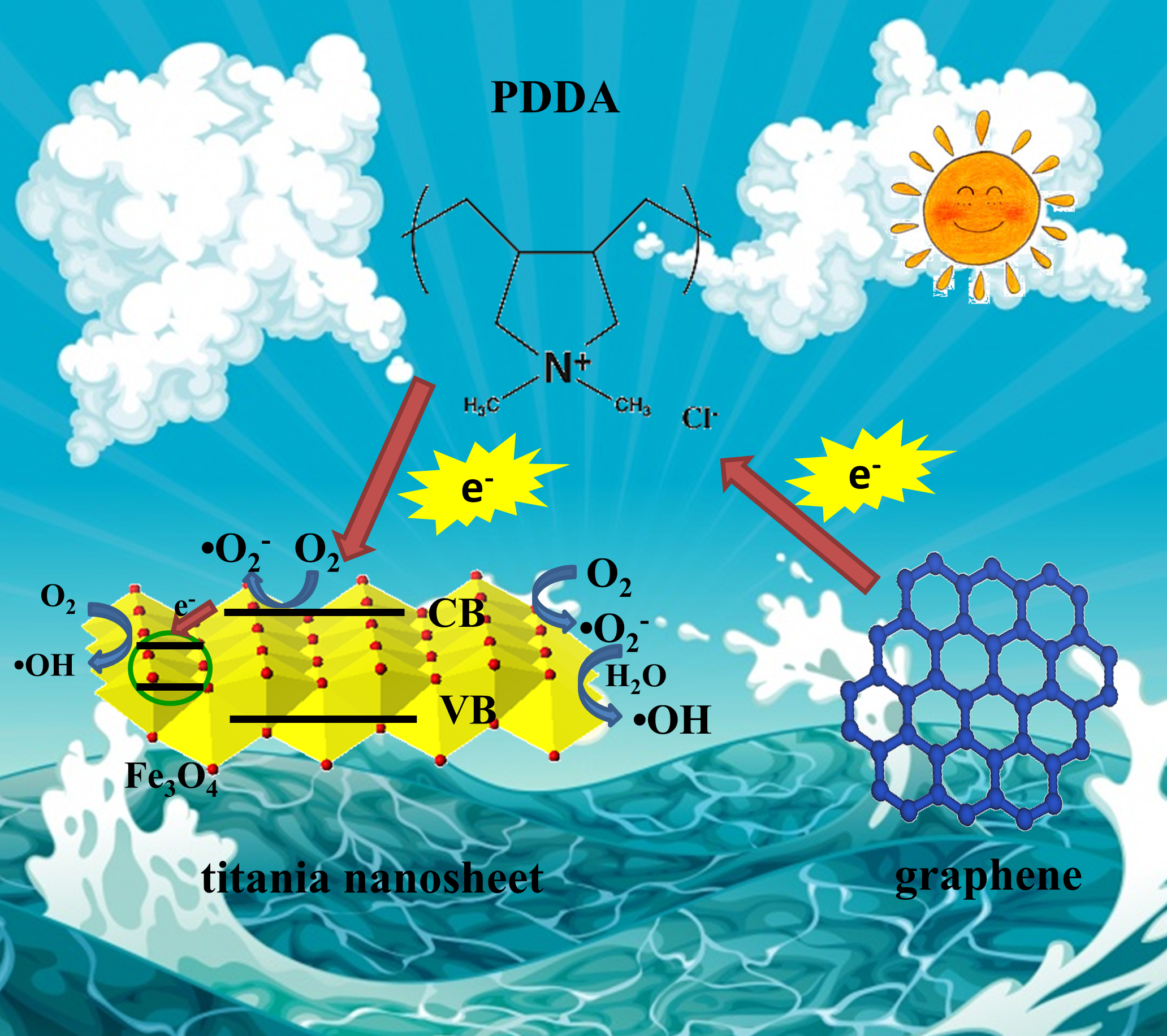


Figure S9. Schematic illustration of charge transfer and photocatalytic processes on the composite under the visible light.

Table S1. The elemental content fractions of the magnetic photocatalysts.

| Element | Wt. % | Atom % |
| --- | --- | --- |
| C K | 59.59 | 69.92 |
| O K | 19.21 | 16.91 |
| N K | 9.97 | 10.03 |
| Ti K | 7.25 | 2.13 |
| Fe K | 3.98 | 1.01 |
